# Supplementary material for: Unraveling the Genetic Diversity and Population Structure of Bangladeshi Indigenous Cattle Populations Using 50K SNP Markers
Source: Animals (Basel). 2021 Aug 12;11(8):2381. doi: 10.3390/ani11082381 (PMC8388693; doi:10.3390/ani11082381)
Supplement: Supplementary file 1 [file animals-11-02381-s001.zip › animals-1318079-SI.pdf]

animals

MDPI

Supplementary Materials

Unraveling the Genetic Diversity and Population Structure of Bangladeshi Indigenous Cattle Populations Using 50K SNP Markers

Mohammad Shamsul Alam Bhuiyan [1], Soo-Hyun Lee [2], Sheikh Mohammad Jahangir Hossain [3], Gautam Kumar Deb [3], Most Farhana Afroz [3], Seung-Hwan Lee [4,*] and Abul Kashem Fazlul Haque Bhuiyan [1,*]

[1] Department of Animal Breeding and Genetics, Bangladesh Agricultural University, Mymensingh 2202, Bangladesh; msabhuiyan.abg@bau.edu.bd
[2] Division of Animal Breeding and Genetics, National Institute of Animal Science, , Cheonan 31000, Korea; lhyungm@korea.kr
[3] Animal Biotechnology Division, Bangladesh Livestock Research Institute, Savar 1341, Bangladesh; smjhossainblri@yahoo.com (S.M.J.H.); debgk2003@yahoo.com (G.K.D.); famukta@yahoo.com (M.F.A.)
[4] Division of Animal and Dairy Science, Chungnam National University, Daejeon 34134, Korea
* Correspondence: slee46@cnu.ac.kr (S.H.L.); bhuiyanbau@gmail.com (A.K.F.H.B.); Tel.: +82-042-821-5878 (S.H.L.); +88-091-67401-6 (ext. 2614) (A.K.F.H.B.)

Citation: Bhuiyan, M.S.A.; Lee, S.-H.; Hossain, S.M.J.; Deb, G.K.; Afroz, M.F.; Lee, S.-H.; Bhuiyan, A.K.F.H. Unraveling the Genetic Diversity and Population Structure of Bangladeshi Indigenous Cattle Populations Using 50K SNP Markers. *Animals* **2021**, *11*, 2381. https://doi.org/10.3390/ani11082381

Academic Editor: Kor Oldenbroek

Received: 13 July 2021
Accepted: 09 August 2021
Published: 12 August 2021

Publisher's Note: MDPI stays neutral with regard to jurisdictional claims in published maps and institutional affiliations.

Copyright: © 2021 by the authors. Licensee MDPI, Basel, Switzerland. This article is an open access article distributed under the terms and conditions of the Creative Commons Attribution (CC BY) license (http://creativecommons.org/licenses/by/4.0/).

Table S1. Cattle breeds or populations and number of samples used in this study.

Sl. No.	Breed/Population	Breed Code	Geographic Location	Sample Size	Type
1.	Sahiwal	SL	Bangladesh	15	Indicine
2.	Munshigani	MC	Bangladesh	26	Indicine
3.	Non-descript Deshi	DES	Bangladesh	22	Indicine
4.	North Bengal Grey	NBG	Bangladesh	21	Indicine
5.	Pabna	PC	Bangladesh	45	Indicine
6.	Red Chittagong	RCC	Bangladesh	89	Indicine
7.	Brahman	BRM	South Asia	25	Indicine
8.	Nellore	NEL	South Asia	21	Indicine
9.	Korean Hanwoo	KPN	East Asia	20	Taurine
10.	Korean Chikso	CHK	East Asia	20	Taurine
11.	Korean Jeju Black	JB	East Asia	60	Taurine
12.	Yianbian	YBH	Central Asia	39	Taurine
13.	Mongol	MG	Central Asia	28	Taurine
14.	Angus	ANG	Europe	24	Taurine
15.	Brown Swiss	BSW	Europe	22	Taurine
16.	Hereford	HFD	Europe	21	Taurine
17.	Holstein	HOL	Europe	30	Taurine
18.	Limousin	LMS	Europe	25	Taurine
19.	Guernsey	GNS	Europe	21	Taurine
20.	Santa geltrudis	SGT	Europe	24	Taurine
21.	Beefmaster	BMA	Europe	24	Taurine
22.	N'Dama	ND	Africa	25	Taurine
23.	Oulmes Zaer	OUL	Africa	26	Taurine
24.	Zebu Madagascar	ZMA	Africa	30	Indicine
25.	Sheko	SHK	Africa	20	Indicine
	**Breed/Population**		**Total sample**	**723**	

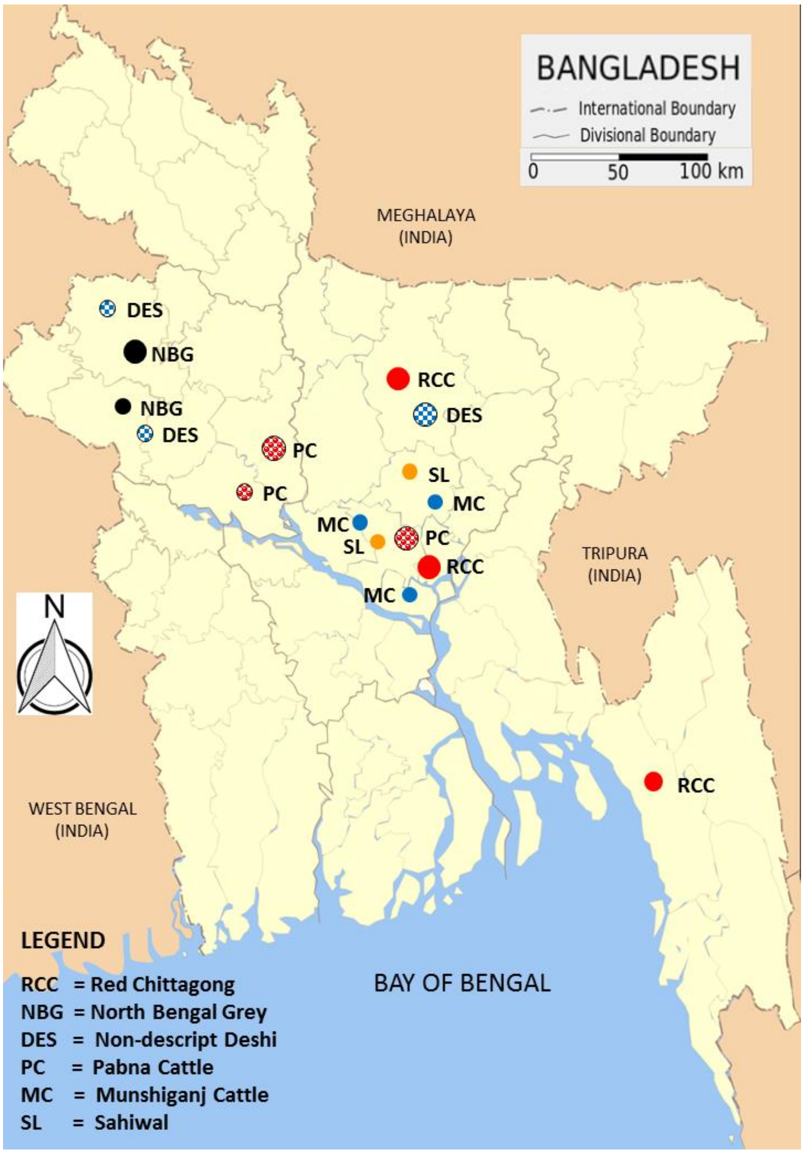

Figure S1. Map of Bangladesh indicating the approximate geographic origin of the cattle samples analysed. Samples of each population were collected from both in-situ and ex-situ conditions. Circle sizes are proportionate with the collected samples.

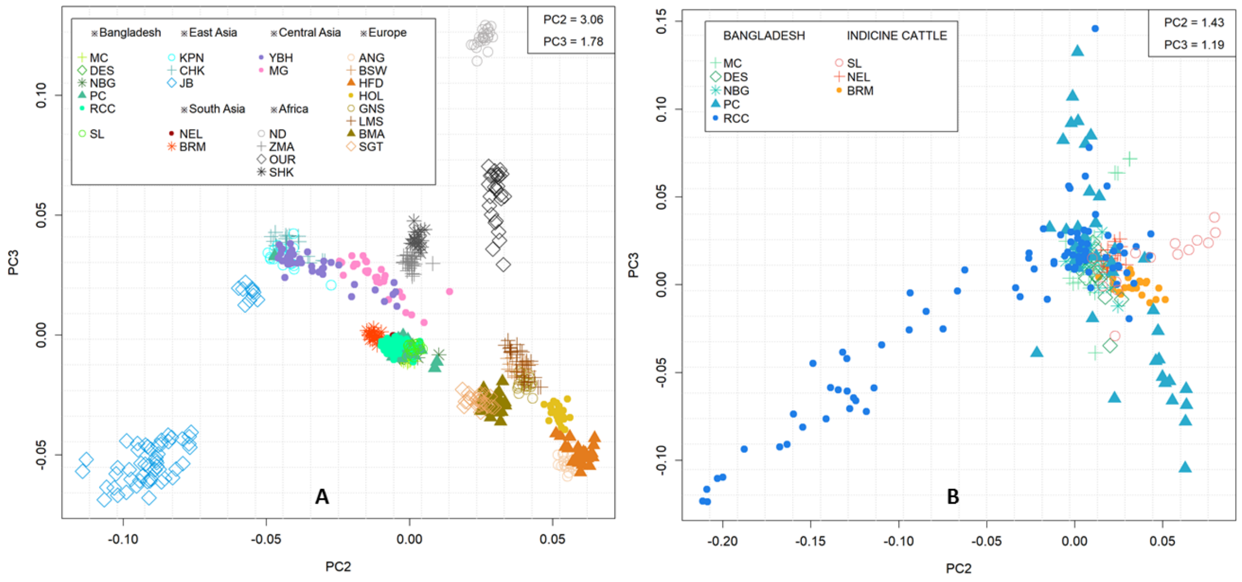

Figure S2. Principal component analysis between PC1 and PC3 in worldwide cattle populations (A) and eight indicine cattle breeds/populations only (B). Cattle populations are labelled as Sahiwal (SL), Munshiganj (MC), Non-descript Deshi (DES), North Bengal Grey (NBG), Pabna (PC), Red Chittagong (RCC), Brahman (BRM), Nellore (NEL), Korean Hanwoo (KPN), Korean Chikso (CHK), Korean Jeju Black (JB), Yianbian (YBH), Mongolian (MG), Angus (ANG), Brown Swiss (BSW), Hereford (HFD), Holstein (HOL), Guernsey (GNS), Limousine (LMS), Santa Gertrudis (SGT), Beefmaster (BMA), N'Dama (ND), Oulmes Zaer (OUR), Zebu Madagascar (ZMA) and Sheko (SHK).

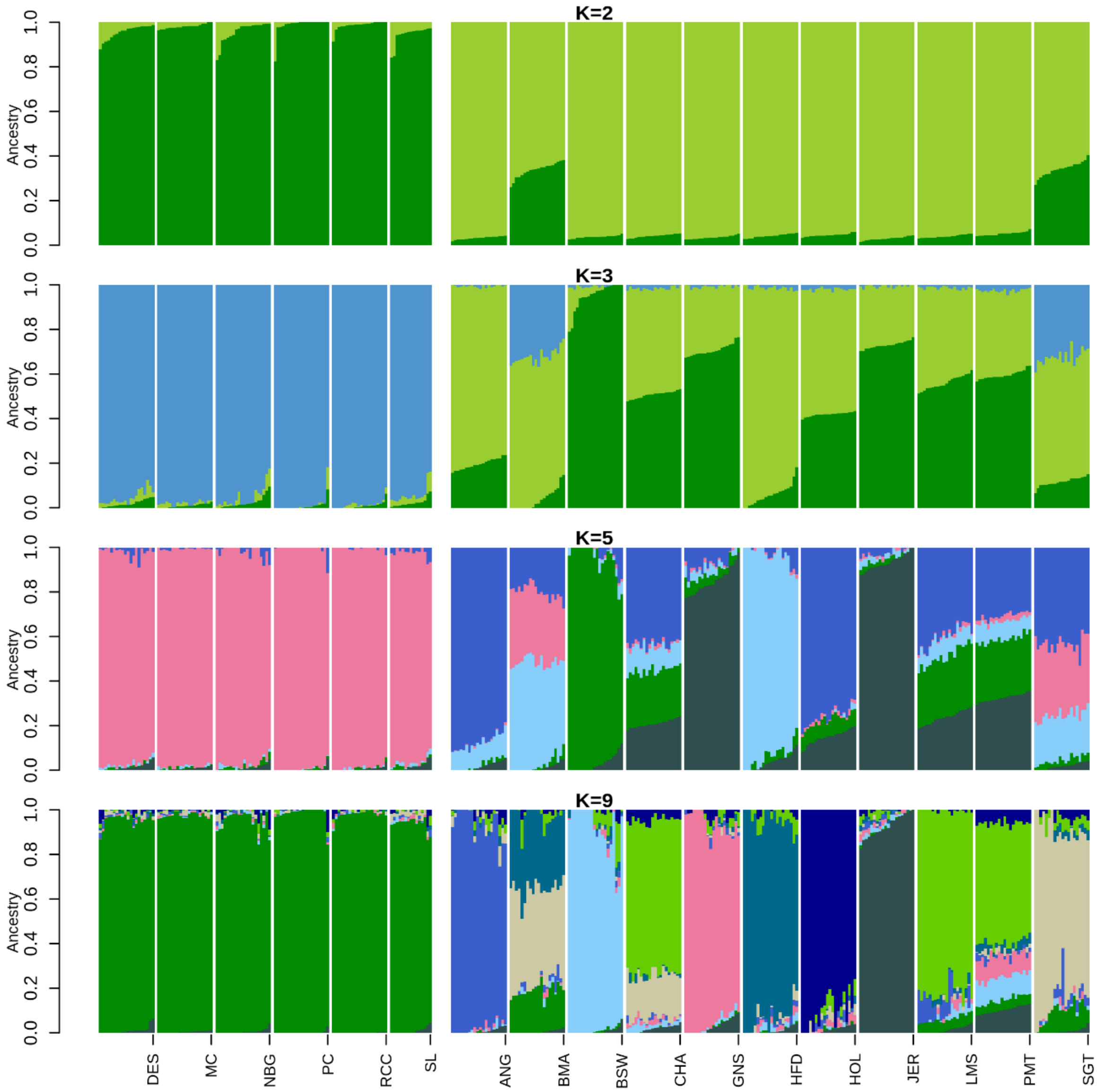

Figure S3. Structure plots showing proportions at assumed ancestries for K = 2 to 9 including 17 breeds/populations from Bangladesh and European taurine breeds. Reference breeds labelled are shown in Figure 2.

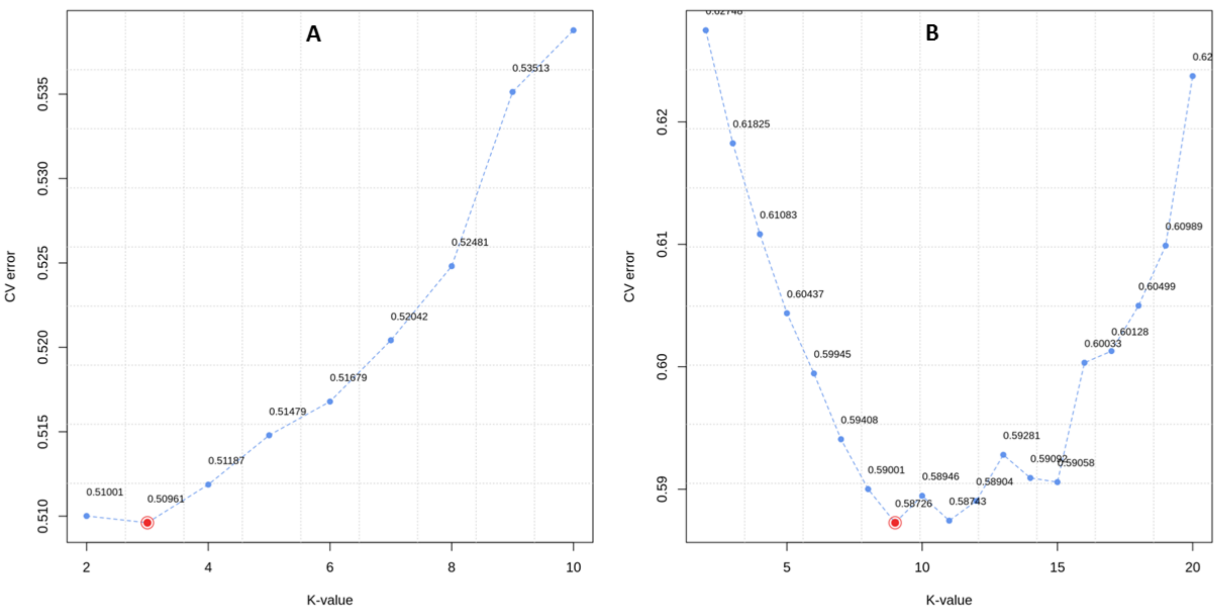

Figure S4. The cross-validation error to determine optimized K value; considering only Indigenous cattle population of Bangladesh (A) and based on 25 cattle populations distributed worldwide (B).
